# MD-GCN: A Multi-Scale Temporal Dual Graph Convolution Network for Traffic Flow Prediction

**DOI:** 10.3390/s23020841

**Published:** 2023-01-11

**Authors:** Xiaohui Huang, Junyang Wang, Yuanchun Lan, Chaojie Jiang, Xinhua Yuan

**Affiliations:** Department of Information Engineering, East China Jiaotong University, Nanchang 330000, China

**Keywords:** traffic flow forecasting, spatial–temporal correlation, graph convolution, temporal convolution

## Abstract

The spatial–temporal prediction of traffic flow is very important for traffic management and planning. The most difficult challenges of traffic flow prediction are the temporal feature extraction and the spatial correlation extraction of nodes. Due to the complex spatial correlation between different roads and the dynamic trend of time patterns, traditional forecasting methods still have limitations in obtaining spatial–temporal correlation, which makes it difficult to extract more valid information. In order to improve the accuracy of the forecasting, this paper proposes a multi-scale temporal dual graph convolution network for traffic flow prediction (MD-GCN). Firstly, we propose a gated temporal convolution based on a channel attention and inception structure to extract multi-scale temporal dependence. Then, aiming at the complexity of the traffic spatial structure, we develop a dual graph convolution module including the graph sampling and aggregation submodule (GraphSAGE) and the mix-hop propagation graph convolution submodule (MGCN) to extract the local correlation and global correlation between neighbor nodes. Finally, extensive experiments are carried out on several public traffic datasets, and the experimental results show that our proposed algorithm outperforms the existing methods.

## 1. Introduction

**Figure 1:**
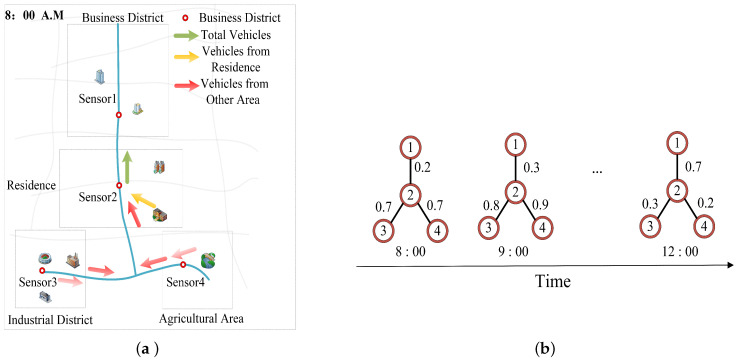
An example of the traffic flow system; (**a**) An example of the traffic flow system in at 8:00 a.m.; (**b**) Dynamic spatial dependency.

With the rapid increase in the number of vehicles in cities, the rational planning of urban transportation has become an important challenge. Intelligent transportation systems (ITS), as a vital intelligent traffic management system in intelligent cities, can provide new solutions to urban road traffic problems. In this paper, we study one of the most representative spatial–temporal forecastings, traffic flow forecasting. Traffic flow is a part of the intelligent transportation system (ITS) [1] and refers to some traffic flow states on the road composed of pedestrians, running vehicles, roads, etc. Traffic flow forecasting uses historical traffic flow data observed by sensors to predict the future [2], which can help people avoid congestion during the journey and choose convenient and safe routes. However, roads in the traffic network have a complex spatial structure. Figure 1 shows a typical traffic system, where traffic sensors are configured at important locations in the road to record traffic flow data. According to Figure 1a, we observe the vehicles beside sensor 2 (green arrow) mainly come from two parts: the first part is the vehicles from the residential area (yellow arrow) adjacent to sensor 2; the second part of flow comes from the two areas: industrial and agricultural vehicles (red arrows), which are relatively far away from sensor 2. The traffic flow within the same road network may change over time, which proves that the spatial dependency is dynamic. An example is shown in Figure 1b; the traffic flow at sensors 3 and 4 can significantly affect the flow of sensor 2 at 8 a.m. and 9 a.m., while there is only a small influence at 12 a.m. and sensor 1 is the opposite of them. We assign different weights to the numbers between the nodes based on the spatial correlation between sensor 2 and its neighbors, and the higher the value, the greater the correlation. Therefore, the spatial and temporal problems caused by these complex traffic structures may bring great challenges to traffic flow prediction.

At present, the problem of traffic flow prediction based on spatio-temporal data has attracted extensive attention from researchers [3,4,5]. In the past few decades, scholars have proposed many methods for predicting traffic flow [6], which includes traditional forecasting models based on statistical methods and predictive models based on machine learning. Among them, the representative one in the traditional prediction model is autoregressive integrated moving average (ARIMA) [7]. However, with the development of society and technology and the capabilities of these models being limited by the stationarity assumption of time series, traditional shallow neural network models are not performing well in the face of increasingly complex transportation networks and huge traffic data volume, and they are usually only applicable to the traffic prediction of a single station. In the face of spatial–temporal data, they cannot extract spatial–temporal correlations well.

At the same time, deep learning has made great breakthroughs in the field of traffic flow prediction [8,9]. For example, convolution neural networks (CNNs) are used to capture the spatial correlation of transportation networks, and recurrent neural networks (RNNs) are used to capture temporal correlations. However, traditional CNNs are often applied to handle the regular grid Euclidean data, and modeling irregular road networks will lose topological information of the traffic network. Graph convolution networks (GCNs) can be used to replace CNNs to better handle non-Euclidean data in traffic road networks [10,11,12]. However, there are still some problems in the graph convolution-based methods. For example, with the deepening of network layers, the graph convolution network will be degraded, and the node information in a longer range cannot be extracted, which leads to the degradation of the prediction performance. The traffic flow often changes periodically, and the traffic flow is also affected by the previous moments. Recurrent neural networks (RNNs) will usually experience time-consuming iterative propagation and gradient bursts when capturing remote time series and often ignore spatial correlations [13].

To address these challenges, we propose a multi-scale temporal dual graph convolution network (MD-GCN). First, we use kernels of different sizes on the temporal convolution module, which can complement the multi-scale temporal dependence and avoid the problem of gradient bursts. After the output of temporal convolution, we use the gating mechanism to filter unnecessary information. From the spatial perspective, as the traffic network becomes more and more complex, the change of traffic flow is obviously affected by its topology, and the traffic flow data between adjacent roads and between roads with a longer range are obviously closely related. However, in previous studies [14,15,16], researchers usually use only one graph convolutional network to build a model and often fail to extract node information in a larger range. In this work, we propose a dual graph convolutiom to extract information at different spatial ranges as well as hidden spatial dependencies between nodes. The main contributions of this paper include the following:

- We propose a dual graph convolution framework with graph sampling and aggregation (GraphSAGE) and mix-hop propagation graph convolution (MGCN) to capture spatial information. By fusing the neighbor nodes information extracted with these two methods, the capability of capturing spatial relations can be further improved.
- We propose a multi-scale temporal convolution with a gated mechanism as a temporal block, in which the temporal correlation of traffic data at different scales is extracted using convolution kernels of different sizes, and the obtained features are fused and adjusted by an efficient pyramid split attention module (EPSA).
- These experimental results conducted on four public datasets show that our proposed algorithm outperforms the existing methods.

The subsequent work of this paper is organized as follows: Section 2 reiviews the works related to traffic prediction. Section 3 introduces the definition of traffic network and problem definition. The framework of the MD-GCN model and the detailed work flow are placed in Section 3. Section 5 verifies the effectiveness of the model through various experiments. Finally, the conclusion and future works are placed in Section 6.

## 2. Related Work

Traffic flow forecasting has long been regarded as an important part of ITS to help alleviate unexpected rising traffic flow, and traffic flow forecasting is a classic time series forecasting task. Compared with the traditional time series and machine learning models, deep learning-based models [12], e.g., Long Short-Term Memory (LSTM) [17] and Gate Recurrent Unit (GRU) [18], show good performance in capturing the temporal correlation of traffic flow data. Meanwhile, the researchers [19] used convolution neural networks and graph neural networks to model spatial correlations. In this section, we summarize the previous traffic flow prediction methods, which mainly include the following two aspects: graph convolution neural network-based models and temporal convolution network-based models [20].

### 2.1. Traffic Prediction Based on Graph Convolution Networks

In recent years, deep learning models have been widely used in traffic flow prediction [21], which mainly includes convolution neural networks (CNNs) and a graph convolution network. In the past, researchers have often used traditional convolution neural networks to model spatial correlations [22,23]. Howerver, due to the complex topology of traffic networks, the results produced by CNN-based methods are usually not satisfactory. Graph convolution neural networks (GCNs) can do well in handling irregular data by integrating the information of neighbor nodes.

Zhao et al. [24] proposed a novel neural network-based traffic forecasting method, the temporal graph convolutional network (T-GCN) model, which is combined with the graph convolutional network (GCN) and the gated recurrent unit (GRU). Li et al. [25] modeled the traffic flow as a diffusion process on a directed graph and introduced a Diffusion Convolutional Recurrent Neural Network (DCRNN) which is able to incorporate both spatial and temporal dependency in the traffic flow prediction. Dai et al. [26] proposed the Hybrid Spatio-Temporal Graph Convolutional Network (H-STGCN), which is able to “deduce” future travel time by exploiting the data of upcoming traffic volume. Lu et al. [27] proposed a spatial–temporal adaptive gated graph convolution network (STAG-GCN) that uses the global context information of roads and spatial–temporal correlation of urban traffic flow to construct a dynamic weighted graph by seeking both spatial neighbors and semantic neighbors of road nodes. Song et al. [28] propose a novel model, named Spatial–Temporal Synchronous Graph Convolutional Networks (STSGCN), for spatial–temporal network data forecasting. The model is able to effectively capture the complex localized spatial–temporal correlations through an elaborately designed spatial–temporal synchronous modeling mechanism. Bai et al. [29] proposed two adaptive modules for enhancing Graph Convolutional Network (GCN) with new capabilities: (1) a Node Adaptive Parameter Learning (NAPL) module to capture node-specific patterns; and (2) a Data Adaptive Graph Generation (DAGG) module to infer the inter-dependencies among different traffic series automatically (AGCRN). Chen et al. [16] proposed the Multi-Range Attentive Bicomponent GCN (MRA-BGCN), which firstly builds the node-wise graph according to the road network distance and the edge-wise graph according to various edge interaction patterns. Guo et al. [30] proposed a novel attention based spatial–temporal graph convolutional network (ASTGCN) model to solve the traffic flow forecasting problem, which mainly consists of the spatial–temporal attention mechanism and the spatial–temporal convolution. Guo et al. [31] proposed a novel Hierarchical Graph Convolution Networks (HGCN) for traffic forecasting by operating on both the micro- and macro-traffic graphs. Wu et al. [15] proposed a novel graph neural network architecture for spatial–temporal graph modeling by developing a novel adaptive dependency matrix and learning it through node embedding, which can precisely capture the hidden spatial dependency in the data. Wu et al. [14] considered the one-way dependency of road and proposed a general graph neural network framework (MTGNN) for multivariate time series data. The model can automatically extract the uni-directed relations among variables through a graph learning module where external knowledge such as variable attributes can be easily integrated.

However, the existing graph convolution models only change the ways of constructing the graph and cannot effectively capture the deep spatial information from the perspective of aggregating nodes. In this work, we design the dual graph convolution module with GraphSAGE [32] and an MGCN module (which use different aggregation methods) to obtain complex feature associations between nodes. In our later experiments, this method is proven to improve the model’s ability to capture spatial information.

### 2.2. Traffic Prediction Based on Temporal Convolution Networks

Recurrent neural networks (RNNs) have often been used for time series prediction. However, traditional RNN-based methods are inefficient when training longer sequences, and their gradients are more likely to explode when combined with graph convolution networks. Therefore, researchers [33,34,35] begin to use Temporal Convolution Networks (TCNs) in traffic flow prediction and achieved better results than RNNs. Yu et al. [33] proposed spatio-temporal graph convolutional networks (STGCN) which prevent the accumulation of errors caused by the iterative training of RNN structures and used temporal convolution networks to extract temporal features on the timeline. In the meantime, Tian et al. [34] proposed spatial–temporal attention wavenet (STAWnet) to handle long time sequences by using TCNs and cature dynamic spatial dependencies between different nodes by using the self-attention network. Li et al. [35] proposed spatial–temporal fusion graph neural networks (STFGNN) to control the input ratio of the original data as the number of network layers increases with the gating mechanism on temporal convolution. However, as the network deepens, the performance of the temporal convolution neural network will deteriorate, since these models cannot extract different ranges of time series information.

## 3. Preliminaries

In this work, we define the traffic topology as G=(V,E,A), where $V=\left\{v_{1},v_{2}\cdots v_{n}\right\}$ represents the set of the sensors on the roads, *E* is the set of edges between nodes representing a connection between two nodes (sensors), the adjacency matrix $A\in R^{n\times n}$ represents the connection relationship between nodes, and *n* is the number of nodes. If there are two nodes $v_{i}$ and $v_{j}$ connecting to each other directly, $A_{ij}$ is set to 1, and it is otherwise set to 0.

We define a feature matrix $X^{t}\in R^{n\times D}$ to represent the traffic flow at time step *t* for all the nodes $V=\left\{v_{1},v_{2}\cdots v_{n}\right\}$, where *D* is the number of traffic features. Given a traffic network graph *G* and the histroical traffic flow, the traffic flow prediction can be defined as a mapping function *f*,

(1) $$\left[X^{\left(t-S:t\right)},G\right]\overset{f}{\to }X^{\left(t+1:t+T\right)},$$

where $X^{\left(t-S:t\right)}\in R^{n\times D\times S}$ is the historical data of *S* time steps and $X^{\left(t+1:t+T\right)}\in R^{n\times D\times T}$ is the traffic flow of *T* time steps to be predicted.

## 4. The Framework of MD-GCN

**Figure 2:**
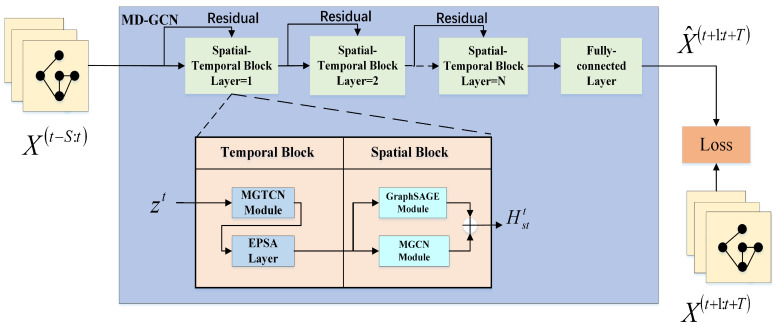
The model structure of MD-GCN.

The structure of MD-GCN presented in this paper is shown in Figure 2. The model mainly includes *N* spatial–temporal blocks and a complete fully connected layer as the output block. In MD-GCN, each spatial–temporal block consists of a spatial block and temporal block. The temporal block is mainly a multi-scale gated temporal convolution module and an efficient pyramid split attention module. The spatial block is composed of a graph sampling and aggregation (GraphSAGE) module and mix-hop propagation graph convolution (MGCN) module. The main innovation of this model is that it constructs modules separately to extract spatial correlation and temporal correlation. For the mining of temporal relations, we use a channel-centered multi-resolution gated temporal convolution model to improve time data processing ability. For the mining of complex spatial relationships, we use the spatial information extracted by the GraphSAGE module and MGCN module to enhance the ability to summarize the information of neighbor nodes. The following sections describe the detailed structure of each module.

### 4.1. Temporal Block

Due to the different traffic conditions at different times in the future, the temporal information extracted by using temporal convolution in TCN [20] is often determined by a fixed convolution kernel. This work introduces the idea of an “inception” structure, using convolution kernels of different sizes to extract time features in different ranges [14]. We propose a multi-scale gated temporal convolution module combined with pyramid channel attention to extract temporal feature information. There are three main processes involved. Firstly, multi-scale gated temporal convolution uses two-dimension convolution to extract temporal correlation. Then, we set convolution kernels of different sizes to improve the range of convolution and use a gated mechanism to filter unnecessary information. Finally, the features obtained are fused and adjusted by the efficient pyramid split attention module and by the channel attention mechanism.

#### 4.1.1. Multi-Scale Gated Temporal Convolution (MGTCN)

**Figure 3:**
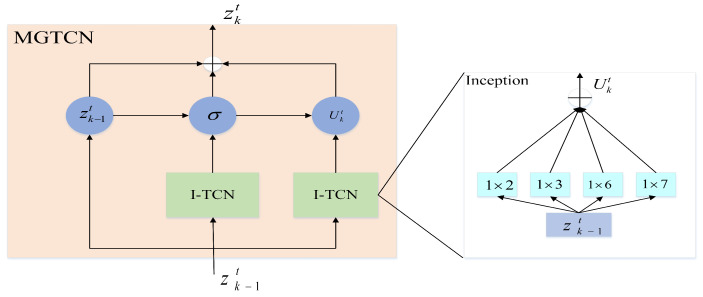
The model structure of MGTCN.

In recent years, the temporal convolution model has been widely used in time series analysis. We propose a multi-scale gated temporal convolution module (MGTCN) as shown in Figure 3. MGTCN mainly includes two parallel multi-scale temporal convolution modules (I-TCN) and a gated fusion module. We define *k* as the number of layers of the current temporal convolution module with k−1 representing its previous layer. The I-TCN module is a temporal convolution module consisting of four different convolution kernels, and the convolution process is defined as:

(2) $$U_{k}^{t}=CONCAT\left(\theta_{k-1}^{1\times 2}*z_{k-1}^{t},\theta_{k-1}^{1\times 3}*z_{k-1}^{t},\theta_{k-1}^{1\times 6}*z_{k-1}^{t},\theta_{k-1}^{1\times 7}*z_{k-1}^{t}\right).$$

$z_{0}^{t}=X^{\left(t-S:t\right)}$, $z_{k-1}^{t}$ is the output of the ${\left(k-1\right)}^{th}$ layer, in which the four filters are truncated to the same length according to the largest filter and concatenated in the channel dimension. $\theta_{k-1}^{1\times 2}$, $\theta_{k-1}^{1\times 3}$, $\theta_{k-1}^{1\times 6}$, $\theta_{k-1}^{1\times 7}$ is the process of convolution using four different convolution kernels, in which 1×2, 1×3, 1×6, and 1×7. ∗ is convolution operation. CONCAT(.) is concatenation operation, and the output after convolution is defined as $U_{k}^{t}$. Then, we use a gated mechanism to filter unnecessary temporal information. The formula is defined as:

(3) $$gated_{k}^{t}=\sigma \left(U_{k}^{t}\times M_{k}+b_{k}\right),$$

(4) $$s_{k}^{t}=\left(1-gated_{k}^{t}\right)*U_{k-1}^{t}+gated_{k}^{t}⊗\left(U_{k}^{t}\times V_{k}+c_{k}\right),$$

$M_{k}$, $V_{k}$, $b_{k}$, $c_{k}$ represent the model parameter of the current layer, ⊗ is the product of elements, and $gated_{k}^{t}$ is the gating coefficient obtained by learning. σ(.) is the Sigmoid function that determines the ratio of information passed to the next layer. The output after temporal convolution and the gated mechanism is defined as $s_{k}^{t}\in R^{n\times F\times C}$, where *F* is the number of time features of the output, and *C* is the number of channels.

#### 4.1.2. Efficient Pyramid Split Attention Module (EPSA)

After MGTCN combines different convolutions by splicing, the channel attention module is introduced to capture the correlation between channels. In this work, we use the efficient pyramid split attention module (EPSA) [32], mainly considering the channel features of different scales on the basis of the previous modules and greatly reducing the complexity of the model on the basis of improving the performance of the deep convolution neural network. First, we focus on the input data $s_{k}^{t}$ cut into *g* parts represented as $s_{k}^{t}{,}_{q}$. The number of channels for each split is $C^{\prime }=\frac{C}{g}$, where $C^{\prime }$ is the number of channels after grouping. Then, we use multi-scale convolution kernels to group convolution, which can reduce the number of parameters. The specific calculation method of multi-scale feature extraction is defined as:

(5) $$F_{k}^{t}{,}_{q}=\mathrm{Conv}\left(K_{q}\times K_{q}\right)\left(s_{k}^{t}{,}_{q}\right),q=0,1,2\ldots g-1,$$

(6) $$F_{k}^{t}=CONCAT\left(\left[F_{k}^{t}{,}_{0},F_{k}^{t}{,}_{1},F_{k}^{t}{,}_{2},\ldots F_{k}^{t}{,}_{g-1}\right]\right),F_{k}^{t}\in R^{n\times S\times C},$$

(7) $$Z_{k}^{t}{,}_{q}=SEWeight\left(F_{k}^{t}{,}_{q}\right),q=0,1,2,\ldots g-1,Z_{k}^{t}{,}_{q}\in R^{1\times 1\times C_{q}},$$

We adaptively select the size of the group according to the size of the convolution kernel, where the relationship between the group and the convolution kernel is $K_{q}=2\times \left(q+1\right)+1$, and Conv(.) represents the process of convolution. $F_{k}^{t}$ is the output obtained by *g* group convolution splicing. We extract channel attention weights for data at different scales by SEWeight(.), $Z_{k}^{t}{,}_{q}$ is the channel attention weight vector of different scales. In order to establish long-term channel attention dependence and to achieve the interaction between multi-scale channel attention, the Softmax function is used here to process the weight parameters, and the formula is defined as:

(8) $$att_{k}^{t}{,}_{q}=Softmax\left(Z_{k}^{t}{,}_{q}\right)=\frac{exp\left(Z_{k}^{t}{,}_{q}\right)}{\sum_{q=0}^{g-1}exp\left(Z_{k}^{t}{,}_{q}\right)},$$

(9) $$z_{k}^{t}{,}_{q}=F_{k}^{t}{,}_{q}⊙att_{k}^{t}{,}_{q},q=1,2,3,\ldots g-1,$$

(10) $$z_{k}^{t}=CONCAT\left(\left[z_{k}^{t}{,}_{0},z_{k}^{t}{,}_{1},\ldots .,z_{k}^{t}{,}_{g-1}\right]\right),$$

where ⊙ is the element-wise product, and $z_{k}^{t}{,}_{q}$ is obtained by multiplying the corresponding eigenvectors $F_{k}^{t}{,}_{q}$ and the weighted coefficients $att_{k}^{t}{,}_{q}$. Finally, the weighted feature vectors are spliced to obtain the output of the temporal module at the $k^{th}$ layer is $z_{k}^{t}$.

### 4.2. Spatial Block

For transportation networks, traffic conditions in adjacent locations influence each other, and the spatial relationship between roads can be captured to predict traffic more accurately. In previous studies, the correlation was usually captured from the global aspect of nodes, and the local correlation of nodes was not fully considered, but transportation networks often contain different dependencies. The spatial module uses the graph sampling and aggregation module and the mix-hop propagation graph convolution module to extract spatial features and hidden spatial dependencies in parallel. The details of the module are defined in the next two sections.

#### 4.2.1. Graph Sampling and Aggregation Module (GraphSAGE)

In this section, we use the GraphSAGE module to spatially model the road network. The module generates node embeddings as follows: given a node $v_{i}\in V$, the set of nodes in its immediate domain is $N(v_{i})$. $h_{l}^{t}{,}_{N(v_{i})}$ is the output of the node $v_{i}$ at the $l^{th}$ layer after aggregating neighbor information. The process of aggregation of all nodes is defined as:

(11) $$h_{l}^{t}{,}_{N(v_{i})}=AGGREGATE\left(h_{l}^{t}{,}_{u},\forall u\in N\left(v_{i}\right)\right),$$

(12) $$h_{l}^{t}{,}_{v_{i}}\leftarrow \sigma \left(W^{l}\cdot MEAN\right)\left(\left\{h_{l-1}^{t}{,}_{v_{i}}\cup \left\{h_{l-1}^{t}{,}_{u},\forall u\in N\left(v_{i}\right)\right\}\right\}\right),$$

(13) $$h_{l}^{t}{,}_{V}=CONCAT\left(h_{l}^{t}{,}_{v_{1}},h_{l}^{t}{,}_{v_{2}},\cdots ,h_{l}^{t}{,}_{v_{n}}\right).$$

$h_{0}^{t}{,}_{V}=z_{out}^{t}\in R^{N\times F\times C}$, $z_{out}^{t}$ is the final output of the temporal block. The current representation of the node $h_{l}^{t}{,}_{v_{i}}$ concatenates with its clustered neighborhood vectors $h_{l-1}^{t}{,}_{u}$ and then feeds into the fully connected layer σ with a nonlinear activation function, which is used for the next presentation. In this work, we use the MEAN(.) aggregator function, and $h_{l}^{t}{,}_{V}$ is the final output at the $l^{th}$ layer.

#### 4.2.2. Mix-Hop Propagation Graph Convolution Module (MGCN)

**Figure 4:**
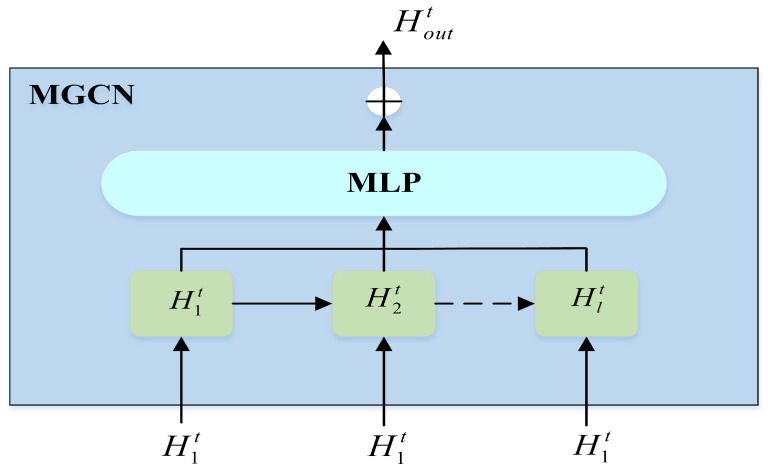
The model structure of MGCN.

In this module, we uses the mix-hop propagation graph convolution module as shown in Figure 4. The MGCN module mainly adopts the mix-hop propagation layer to handle information flow on spatially related nodes, which consists of two steps, information propagation and information selection. The module can preserve the original state of some nodes in the process of propagation so that the state of the propagated nodes can not only maintain the locality but also explore the deep neighborhood. Given G=(V,E,A), the information propagation is defined as:

(14) $$H_{l}^{t}=\mu H_{1}^{t}+\left(1-\mu \right)\tilde{A}H_{l-1}^{t},$$

μ is a hyperparameter mainly used to control the proportion of the original node state, $H_{l}^{t}$ and $H_{l-1}^{t}$ represent the output of the $l^{th}$ layer and ${\left(l-1\right)}^{th}$ layer, $H_{1}$ represents the output of the previous layer, and $H_{1}^{t}=z_{out}^{t}$ for the normalized adjacency matrix. The information selection step is defined as follows:

(15) $$H_{\mathrm{out}\;}^{t}=\sum_{l=1}^{L}H_{l}^{t}W_{l},$$

*L* is number of layers for graph convolution, and $H_{\mathrm{out}\;}^{t}$ represents the current layer output. The parametric matrix $W^{l}$ is used as a feature selector, and we set the value to zero when the graph structure does not have a spatial dependency to preserve the original structure information.

(16) $$H_{st}^{t}=h_{out}^{t}{,}_{V}⊕H_{\mathrm{out}\;}^{t},$$

$h_{out}^{t}$ is the final output of the GraphSAGE module, and ⊕ is the addition of elements. The structure of the double-graph convolution is added to obtain the output of the temporal and spatial module $H_{st}^{t}$.

## 5. Experiments

In this section, we verify the effectiveness of our proposed model on four real datasets. We will introduce the experiments in detail from the aspects of experiment setup, baselines, convergence analysis, parameter study, experiment results, ablation experiment, and case study.

### 5.1. Experiment Setup

#### 5.1.1. Dataset

We evaluate the preformace of our proposed model and baseline models on four widely used traffic datasets. The properties of the datasets are summarized in Table 1. Traffic speed and traffic flow are both important research questions for traffic forecasting, and we collected two representative datasets. METR-LA and PEMS-BAY are traffic speed datasets. PEMS04 and PEMS08 are traffic flow datasets. Nodes represent the number of sensors on the traffic network and Edges are weights, which are obtained by the distance between sensors on the traffic network. The data collection interval is every five minutes as a time step. Because of the speed limitations of these regions, traffic speed is floating-point data and traffic flow data represent the number of passing vehicles.

- METR-LA [14,15]: It is a public traffic speed dataset collected from Los Angeles County highways that contains data from 207 sensors from 1 March 2012 to 30 June 2012. Sensors are used to detect the presence or passage of vehicles, mainly detecting traffic information, including traffic flow and traffic speed information. Traffic speed is recorded every five minutes for a total of 34,272 time slices.
- PEMS-BAY [14,15]: It is a dataset of public traffic speeds collected from the California Department of Transportation measurement system. Specifically, PEMS-BAY contains data from 325 sensors in the Gulf over a six-month period from 1 January 2017 to 31 May 2017. Traffic information is recorded at a rate of 5 min with a total 52,116 time slices.
- PEMS04 [28,35]: It is a dataset of public traffic flows collected from CalTrans PeMS. Specifically, PEMS04 contains data from 307 sensors in District 04 over a two-month period from 1 January 2018 to 28 February 2018. Traffic information is recorded every 5 min, and the total number of time slices is 16,992.
- PEMS08 [28,35]: It is a dataset of public traffic flow collected from CalTrans PeMS. Specifically, PEMS08 contains data from 170 sensors in District 08 for a two-month period from 1 July 2018 to 31 August 2018. Traffic information is recorded every 5 min, and the total number of time slices is 17,856.

#### 5.1.2. Parameter Setting

We divided the dataset into a training set, validation set, and testing set in the ratio of 7:1:2 and used the same hyperparameters on four datasets. *S* and *T* are set equal to 12, the first *S* time steps are our input data, and the last *T* time steps are considered to be our actual label values. Using 12 consecutive time steps from the past, we predicted 12 successive time steps in the future. In each dataset, all experiments were repeated ten times. The number of layers *N* for the entire spatial–temporal block is set to 3; the number of layers *L* of the spatial blcok is set to 2; and the number of layers *K* of the temporal block is set to 3. In the model proposed in this paper, all the convolution operations are set with 64 filters (including graph convolution and 1D convolutional network). In the spatial–temporal block, the size of the hidden layers was set to 64. The initial value of the expansion factor was set to 2. In the training stage, we use adam to optimize the model, the batch size is 32, and the learning rate is set as 0.001. Table 2 provides a detailed description of the parameter setting.

#### 5.1.3. Evaluation Function

We use three evaluation metrics commonly used in baseline papers to evaluate the predictive effect of the model, including mean absolute error (MAE), root mean square error (RMSE), and mean absolute percentage error (MAPE). The formula is shown below:

(17) $$\mathrm{RMSE}=\sqrt{\frac{1}{R}\sum_{r=1}^{R}{\left(X_{r}^{\left(t+1:t+T\right)}-{\hat{X_{r}}}^{\left(t+1:t+T\right)}\right)}^{2}},$$

(18) $$\mathrm{MAE}=\frac{1}{R}\sum_{r=1}^{R}\left|(X_{r}^{\left(t+1:t+T\right)}-{\hat{X_{r}}}^{\left(t+1:t+T\right)})\right|,$$

(19) $$\mathrm{MAPE}=\sum_{r=1}^{R}\left|\frac{X_{r}^{\left(t+1:t+T\right)}-{\hat{X_{r}}}^{\left(t+1:t+T\right)}}{X_{r}^{\left(t+1:t+T\right)}}\right|\times \frac{100}{r},$$

among them, the MAE measure reflects the prediction accuracy, the RMSE is more sensitive to outliers, and MAPE can eliminate the influence of data units to a certain extent. *R* is the total number of samples, and $X_{r}^{\left(t+1:t+T\right)}$ and ${\hat{X_{r}}}^{\left(t+1:t+T\right)}$ are the actual and predicted values of the $r^{th}$ sample. The smaller the value of the above metrics, the better the predictive performance of the model.

### 5.2. Baselines

We selected the latest research methods to compare our models.

- FC-LSTM [17]: This model uses a Long Short-Term Memory network with fully connected hidden cells to predict traffic data.
- T-GCN [24]: This model uses, respectively, GCN and GRU to capture the spatial and temporal correlations of transportation networks.
- Graph WaveNet [15]: This model introduces a self-adaptive graph to capture the hidden spatial dependency and uses dilated convolution to capture the temporal dependency.
- STFGNN [35]: This model uses spatial–temporal graphs to capture spatial–temporal correlations in traffic networks.
- STSGCN [28]: This model uses a spatial–temporal synchronous graph convolution network to independently model local correlations through a local time–space subgraph module.
- DCRNN [25]: This model uses a diffusion–convolution recursive neural network, which combines diffusion graph convolution with a recurrent neural network.
- STGCN [33]: The model combines graph convolution with one-dimensional convolution to capture spatial–temporal correlations.
- ASTGCN [30]: This model uses a spatial–temporal attention mechanism to capture the dynamic spatial–temporal characteristics of traffic data.
- MTGNN [14]: This is a multi-variable time series prediction model using a graph neural network from a graph perspective.

### 5.3. Convergence Analysis

**Figure 5:**
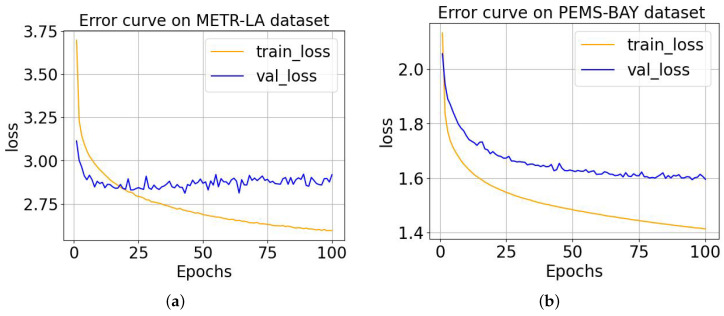
The training and validation error curves of the MD-GCN model on two datasets. (**a**) MD-GCN training and validating errors on the METR-LA dataset; (**b**) MD-GCN training and validating errors on the PEMS-BAY dataset.

**Figure 6:**
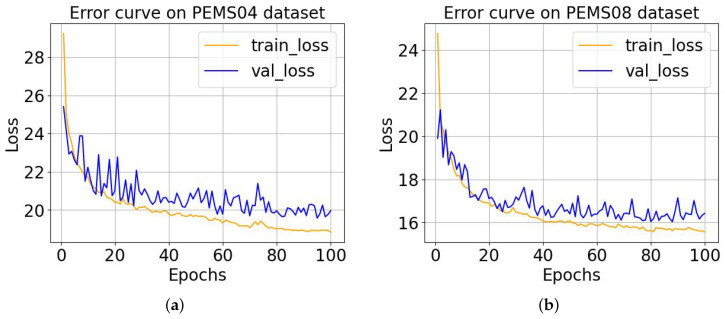
The training and validation error curves of the MD-GCN model on two datasets. (**a**) MD-GCN training and validating errors on the PEMS04 dataset; (**b**) MD-GCN training and validating errors on the PEMS08 dataset.

In order to explore the convergence of our proposed model, we show the error between the ground truth and the prediction results preduced by MD-GCN in the training and validation process on the four datasets in Figure 5 and Figure 6. The X-axis in the figures represents the number of training epoches, and the Y-axis represents the loss of the training process and validation. We can see that as the number of training epoches increases, the loss continues to decrease and eventually reaches a convergent state. It can be seen that the results of the training and validation losses tend to stabilize after 80 epoches, which indicates that the model has reached the convergence state. The remaining three datasets can also converge after 80 epoches from Figure 5 and Figure 6. Therefore, in a later study, we set the number of training epoches to 100 (slightly greater than 100).

### 5.4. Parameters Study

**Figure 7:**
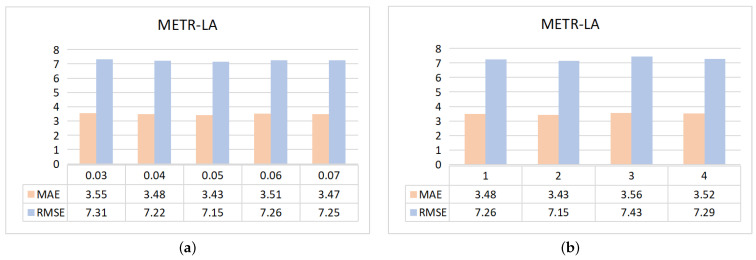
Study of model parameters on METR-LA; (**a**) the error of the parameter λ at different values; (**b**) errors at different values of the number of layers of the spatial block.

In the section, Figure 7 shows our study of two parameters in our model on the dataset METR-LA parameters; the X-axis represents the set value of the parameter, and the Y-axis represents the two evaluation indicators of MAE and RMSE.

In the spatial block, as the number of network layers deepens, node representations of the same connectivity graph tend to have the same value; it is impossible to distinguish between different nodes (over-smoothing). In order to solve the problem, we set an initial node information retention factor λ. As shown in Figure 7a, the values of the parameters are set to $\left[0.03,0.04,0.05,0.06,0.07\right]$; when λ takes 0.05, the experimental error is minimal.

The number of layers in the spatial block will have different effects on the extraction of spatial information, so we use an experimental comparison to select the most suitable number of layers for the spatial block. As shown in Figure 7b, the number of layers is set to four values, $\left[1,2,3,4\right]$; when the number of layers is taken by 2, the experiments predict the best results.

### 5.5. Experimental Results

Table 3 and Table 4 show the experimental results of our proposed model compared with different baselines on METR-LA and PMES-BAY. Horizon 3, 6, and 12 represent the third, sixth, and twelfth time steps, respectively, representing 15 min, 30 min and 60 min to predict the situation. The results show that our proposed model consistently outperforms the baselines on the METR-LA and PMES-BAY datasets, especially on the predictions of 30 min and 60 min. This reason may be that convolution-based approaches are less able to capture more spatial dependencies, whereas our dual graph convolution can capture more hidden spatial dependencies and features, thus improving the prediction results. Compared with MTGNN, our model reduced MAE and RMSE by 2.01%, 2.81%, 1.71%, and 2.11% at 30 min and 60 min on the METR-LA dataset. In Table 5, we compared the results produced by different models on the PEMS04 and PMES08 datasets with repsect to MAE, RMSE and MAPE. Compared with the model STFGNN, our model improved by 6.53%, 7.63%, and 3.33% on three evaluation metrics, respectively, on PEMS08. MD-GCN also achieved better results than the baselines on other datasets. This reason may be that the multi-scale gated temporal convolution module can capture temporal correlation over different time periods and achieve better results on the average prediction results.

**Figure 8:**
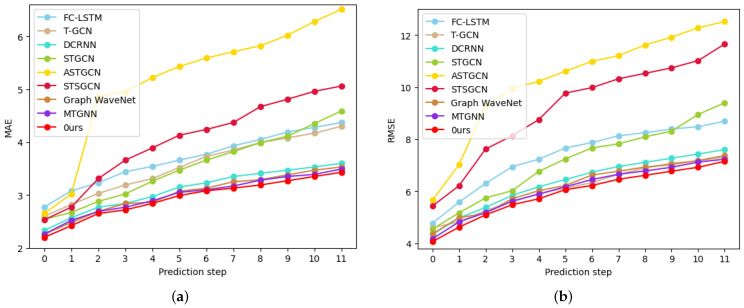
Comparison of each step error of all models on dataset METR-LA: (**a**) MAE; (**b**) RMSE.

**Figure 9:**
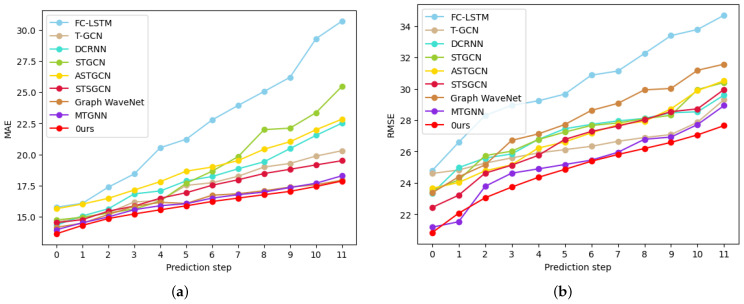
Comparison of each step error of all models on dataset PEMS08: (**a**) MAE; (**b**) RMSE.

Compared with ASTGCN, STFGNN, MTGNN, and GraphWaveNet, the MD-GCN model proposed in this paper adopts the method of constructing spatial–temporal information mining hidden structures. In a temporal block, we use channel attention mechanisms and temporal convolution networks to combine the characteristics of data at different scales. Our spatial block adopts the method of graph convolution and graph aggregation sampling dual graph fusion to integrate the spatial information extracted in different ways. To further investigate the effect of our model, we show the training error at each time step of the two datasets METR-LA and PMES08 in Figure 8 and Figure 9; our model performed better than the other models at each step of these two data. FC-LSTM and T-GCN perform the worst; as the length of the prediction increases, the prediction performance decreases significantly, which proves the validity of the spatial–temporal blocks. DCRNN, STGCN, ASTGCN, and GraphWaveNet have similar predictive performance and can all achieve good results in short-term time steps. However, the stability of these models is not enough, and the performance degradation rate is significantly higher than that of our model. Although the most stable of these comparison models is MTGNN, MTGNN is weaker than our overall prediction accuracy. Our model predicts significantly more stable curves and slower performance degradation.

### 5.6. Ablation Experiments

In order to verify the effectiveness of each module in the model, we performed ablation tests on four datasets, and the main process is as follows:

- w/o GraphSAGE: In the mixed hop propagation graph convolution module, we remove the GraphSAGE module.
- w/o EPSALayer: In the temporal module, we remove the efficient pyramid split attention module.
- w/o MGTCN: We replace the multi-scale gated temporal convolution module with a normal time convolution module.

**Figure 10:**
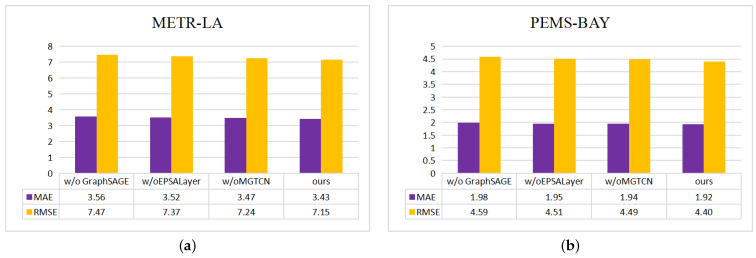
Experimental results of different ablation modules: (**a**) METR-LA; (**b**) PMES-BAY.

**Figure 11:**
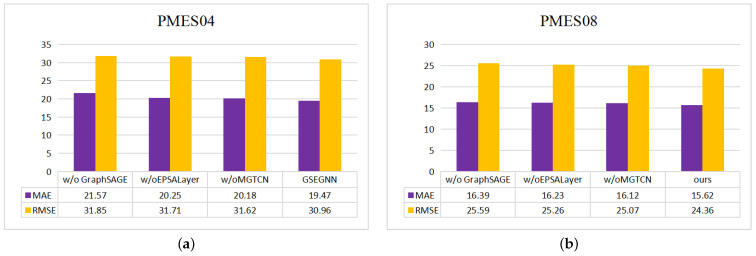
Experimental results of different ablation modules: (**a**) PMES04; (**b**) PMES08.

In our experimental setup, we first verify the validity of the dual graph convolution module and then use the graph convolution module alone to extract the spatial structure information. Second, we validated the need for the channel focus mechanism by removing the EPSAlayer module. Finally, we choose the traditional temporal convolution module to verify the MGTCN module. As shown in Figure 10 and Figure 11, the GraphSAGE module plays a key role in the model, and the other two modules on our model also play a different role. Thus, the validity of the various modules in our MD-GCN model is verified.

### 5.7. A Case Study

**Figure 12:**
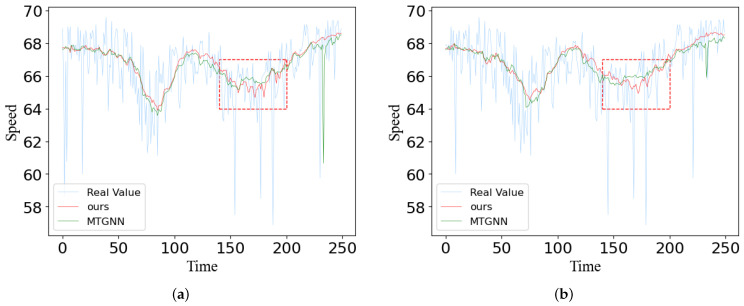
Traffic speed case study of two different stations on the METR-LA dataset: (**a**) sensor 1; (**b**) sensor 2.

**Figure 13:**
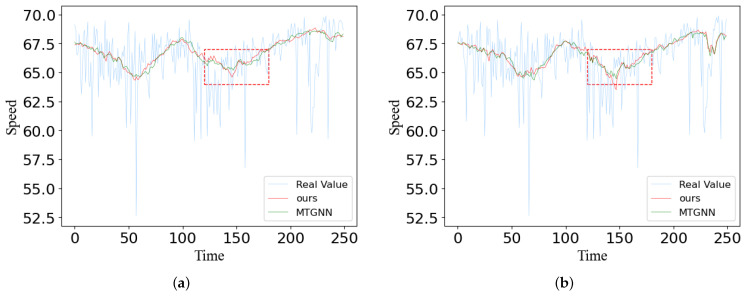
Traffic speed case study of two different stations on the PEMS-BAY dataset: (**a**) sensor 1; (**b**) sensor 2.

In this section, we plotted the predictions of MTGNN and our model 60 min ahead against the actual values on both datasets. We randomly selected the prediction of two sensors over time from two datasets, and the final result is shown in Figure 12 and Figure 13. The X-axis represents the number of time steps and the Y-axis is the traffic speed at which the vehicle is traveling. Sensor 1 and sensor 2 are the two adjacent sensors we selected. We obtain some conclusions by observation figures: (1) with the change of time, when the true value of traffic oscillates, our predicted value generates a smoothed prediction of the average, reflecting the robustness of our model; (2) for spatial relationships, the predictions of two adjacent sensors tend to show similar characteristics; (3) as shown by the red dotted line in the figures, in the face of sudden changes in traffic speed, our model predicts more accurate results than MTGNN; (4) due to the different patterns of different geographical locations, the congestion time periods reflected on the two figures are not exactly consistent, but our model can capture hidden dependencies between nodes and can represent good stability and performance in spatial–temporal prediction. The prediction curve of our model can match the true flow curve better than Graph Wavenet, which further verifies the necessity of using the mode of dual graph convolution to extract multi-range spatial features and multi-scale gated convolution to extract richer temporal features.

### 5.8. Discussion

From the experimental results, we can see that our proposed MD-GCN model is able to obtain performance improvements in terms of the evaluation metrics: RMSE, MAE, and MAPE. Compared with our dual graph convolution module, MTGNN and Graph WaveNet only use adaptive graph convolution to extract spatial features, which makes it difficult to show good results in both long-term and short-term prediction. Our proposed model can enhance the ability to extract hidden spatial information by integrating two graph convolution methods to aggregate node information of different ranges. Compared with our MGTCN module and EPSA module in a temporal block, STSGCN and STFGNN use the temporal convolution to extract time information, and the predictions on average time steps are also not as effective as our model. Our proposed temporal module can extract time features at different ranges and adjust the features using channel attention to obtain more effective time correlation. From the results on these representative evaluation metrics, our model shows more stable and better results in traffic flow prediction than these popular baselines.

From the results obtained by the ablation experiment, we can find that our proposed dual graph convolution module and multi-scale gated temporal convolution module, as well as the EPSA module, can improve the accuracy of prediction, which also explains the necessity of our work. From the comparison of real road data and forecast data in the case study, we can intuitively observe that our model shows better stability and accuracy in the face of complex traffic data than other baseline models.

## 6. Conclusions

In this paper, we propose a novel spatial–temporal model (MD-GCN) to predict traffic conditions. Specifically, in terms of time dependence, we propose a gated temporal convolution module based on multi-scale channel attention combined with an “inception” structure. By expanding the width of the convolution network and combining the receptive field of temporal convolution at different scales, the temporal relationship capture ability of the model is effectively improved. For spatial dependencies, we combine two modules: the GraphSAGE module and the mix-hop propagation graph convolution module. The spatial information extracted by fusing the two modules improves the ability of the model to obtain feature relationships of different ranges in traffic networks. Finally, we choose to verify the validity and stability of the model on four datasets METR-LA, PEMS-BAY, PEMS04, and PEMS08. In addition, the ablation experiments again validate the effectiveness of our model. For future work, we will consider the influence of various external factors to further improve our work.

## Figures and Tables

**Table 1:** The details of the datasets.

Type	Dataset	Sensor (Nodes)	Edges	Time Step
Speed	METR-LA	207	1722	34,272
Speed	PMES-BAY	325	2694	52,116
Flow	PEMS04	307	680	16,992
Flow	PEMS08	170	548	17,856

**Table 2:** The details of the parameter setting.

Parameters	Value
Input length (S)	12
Output length (T)	12
Spatial–temporal block (N)	3
Temporal block (K)	3
Spatial block (L)	2
Hidden layers	64
Batch Size	32
Optimizer	adam

**Table 3:** The comparative results on METR-LA.

	Horizon 3			Horizon 6			Horizon 12		
Method	MAE	RMSE	MAPE (%)	MAE	RMSE	MAPE (%)	MAE	RMSE	MAPE (%)
FC-LSTM	3.44	6.30	9.60	3.77	7.23	10.09	4.37	8.69	14.00
T-GCN	3.03	5.26	7.81	3.52	6.12	9.45	4.30	7.31	11.80
DCRNN	2.77	5.38	7.30	3.15	6.45	8.80	3.60	7.60	10.50
STGCN	2.88	5.74	9.21	3.47	7.24	9.57	4.59	9.40	12.70
ASTGCN	4.86	9.27	7.81	5.43	10.61	10.13	6.51	12.52	11.64
STSGCN	3.31	7.62	8.06	4.13	9.77	10.29	5.06	11.66	12.91
Graph WaveNet	2.69	5.15	6.90	3.07	6.22	8.37	3.53	7.37	10.01
MTGNN	2.69	5.18	6.86	3.05	6.17	8.19	3.49	7.23	9.87
MD-GCN (Ours)	2.65	5.09	6.82	2.99	6.06	8.19	3.43	7.15	10.04

**Table 4:** The comparative results on PEMS-BAY.

	Horizon 3			Horizon 6			Horizon 12		
Method	MAE	RMSE	MAPE (%)	MAE	RMSE	MAPE (%)	MAE	RMSE	MAPE (%)
FC-LSTM	2.05	4.19	4.80	2.20	4.55	5.20	2.37	4.96	5.70
T-GCN	1.50	2.83	3.14	1.73	3.40	3.76	2.18	4.35	4.94
DCRNN	1.38	2.95	2.90	1.74	3.97	3.90	2.07	4.74	4.90
STGCN	1.36	2.96	2.90	1.81	4.27	4.17	2.49	5.69	5.79
ASTGCN	1.52	3.13	3.22	2.01	4.27	4.28	2.61	5.42	6.00
STSGCN	1.44	3.01	3.04	1.83	4.18	4.17	2.26	5.21	5.40
Graph WaveNet	1.30	2.74	2.73	1.63	3.70	3.67	1.95	4.52	4.63
MTGNN	1.32	2.79	2.77	1.65	3.74	3.69	1.94	4.49	4.53
MD-GCN(Ours)	1.32	2.81	2.77	1.64	2.71	3.66	1.92	4.40	4.45

**Table 5:** The comparative results on PEMS04 and PEMS08.

	PMES04 (Mean)			PMES08 (Mean)		
Method	MAE	RMSE	MAPE (%)	MAE	RMSE	MAPE (%)
FC-LSTM	27.14	41.59	18.20	2.20	22.20	34.06
T-GCN	21.34	32.35	14.42	17.86	26.12	10.76
DCRNN	22.16	34.22	14.83	17.86	27.83	11.45
STGCN	22.70	35.55	14.59	18.02	27.83	11.40
ASTGCN	22.93	35.22	16.56	18.61	28.16	13.08
STSGCN	21.19	33.65	13.90	17.13	26.80	10.96
Graph WaveNet	25.45	39.70	17.29	19.83	31.05	12.68
STFGNN	19.83	31.88	13.02	16.64	26.22	10.60
MTGNN	19.90	31.73	13.46	16.55	25.48	10.50
MD-GCN(Ours)	19.47	30.96	13.33	15.62	24.36	10.26

## Data Availability

The public datasets METR-LA and PEMS-BAY can be obtained at https://github.com/liyaguang/DCRNN. PMES04 dataset and PEMS08 dataset can be obtained at https://github.com/MengzhangLI/STFGNN.
